# Screening and identification of a non-peptide antagonist for the peptide hormone receptor in *Arabidopsis*

**DOI:** 10.1038/s42003-019-0307-8

**Published:** 2019-02-15

**Authors:** Hidefumi Shinohara, Naoko Yasue, Tetsuo Onuki, Yasumitsu Kondoh, Minoru Yoshida, Yoshikatsu Matsubayashi

**Affiliations:** 1 Division of Biological Science, Graduate School of Science, Nagoya University, Chikusa, Nagoya, 464-8602 Japan; 2 National Institute for Basic Biology, Myodaiji, Okazaki, 444-8585 Japan; 3 RIKEN Center for Sustainable Resource Science, Hirosawa 2-1, Wako, 351-0198 Japan

## Abstract

Intercellular signaling mediated by peptide hormones and membrane-localized receptor kinases plays crucial roles in plant developmental processes. Because of their diverse functions, agonistic or antagonistic modulation of peptide signaling holds enormous promise for agricultural applications. Here we established a high-throughput screening system using a bead-immobilized receptor kinase and fluorescent-labeled peptide ligand to identify small molecules that bind peptide hormone receptors in competition with natural ligands. We used the *Arabidopsis* CLE9-BAM1 ligand-receptor pair to screen a library of ≈30,000 chemicals and identified NPD12704 as an antagonist for BAM1. NPD12704 also inhibited CLV3 binding to BAM1 but only minimally interfered with CLV3 binding to CLV1, the closest homolog of BAM1, demonstrating preferential receptor specificity. Treatment of *clv1-101* mutant seedlings with NPD12704 enhanced the enlarged shoot apical meristem phenotype. Our results provide a technological framework enabling high-throughput identification of small non-peptide chemicals that specifically control receptor kinase–mediated peptide hormone signaling in plants.

## Abstract (web summary)

Hidefumi Shinohara and colleagues used the CLE9-BAM1 ligand-receptor pair as a model system for screening peptide hormone receptor-binding small molecules in plants. They identified the small molecule NPD12704 as an antagonist for BAM1 and demonstrated the specific regulatory activity of NPD12704 in shoot apical meristem.

## Introduction

Extracellular signaling mediated by small peptide hormones and membrane-spanning receptor kinases plays crucial roles in numerous developmental processes in plants, including vegetative growth, stem cell regulation, vascular differentiation, nitrogen acquisition, pollen tube guidance, tissue abscission, symbiosis regulation, stomata differentiation, and diffusion barrier formation^1–4^. Most of these peptides act as local signaling mediators of proximal cell-to-cell communication, whereas others mediate long-distance mobile signaling required for tissue-to-tissue communication. Peptide hormones in plants appear to be functionally more diverse than previously thought^1–4^.

Because of the diverse and specific functions of peptide hormones, artificial modulation of peptide signaling pathways holds great promise for agricultural applications. Most peptides, however, penetrate poorly into plant tissues, especially the above-ground parts covered by the waterproofing cuticle. The cuticular penetrability of an exogenously applied molecule correlates with its lipophilicity, as indicated by the wax/water partition coefficient^5^. Another problem that limits the practical application of peptides is their proteolytic instability in planta and in the microbe-rich natural soil environment. A major translational challenge that must be met in attempting to overcome these limitations is development of non-peptide agonists/antagonists that specifically activate/block peptide hormone receptors. Historically, small molecules that act as agonists or antagonists for classical plant hormones such as auxin, cytokinin, and abscisic acid have been used both in fundamental mechanistic research and agricultural applications to control hormonal effects^6^. To date, however, no such chemicals have been reported for peptide hormone signaling in plants.

In mammalian cells, G protein–coupled receptors (GPCRs) are the largest and most versatile group of cell surface receptors for peptide hormones, and accordingly, they have become major targets for drug discovery^7^. For GPCR-targeted chemical screening, measurement of intracellular levels of secondary messengers such as cAMP, inositol phosphate, and calcium have often been employed as common readouts of receptor activation because these molecules play a shared role in GPCR-induced signaling^8^. However, except for several pathogen-recognizing receptors^9^, no common readout has been reported for plant receptor kinase signaling, which makes it difficult to screen chemicals using conventional cell-based assays in plants.

In this study, we established a high-throughput binding assay-based screening system using a bead-immobilized receptor kinase^10^ and fluorescent-labeled peptide ligand to identify small molecules that bind peptide hormone receptors in competition with natural peptide ligands. We used *Arabidopsis* receptor kinase BAM1^11^ as a model, primarily because this receptor kinase plays a pivotal role in regulating shoot apical meristem (SAM) size redundantly with the closely related receptor kinase CLV1^12^ by recognizing the peptide ligand CLV3^13–16^. BAM1 also interacts with several CLV3 homologs with high affinity, including CLE9 peptide, which enabled us to synthesize a high-affinity fluorescent-labeled ligand by introducing a fluorescent group into evolutionarily unconserved residues^17^. Using this system, we screened a library of ~30,000 chemicals and identified one compound that acts as an antagonist for BAM1.

## Results

### Visualization of the CLE9–BAM1 interaction on microbeads

**Fig. 1:**
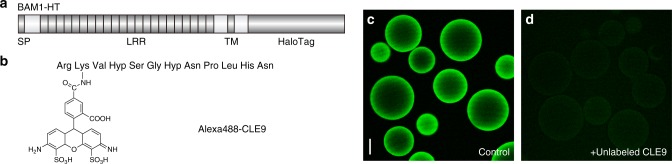
Microscopic visualization of the CLE9–BAM1 interaction on microbeads. **a** Structure of recombinant BAM1, in which the cytoplasmic kinase domain was replaced by HaloTag (BAM1-HT). BAM1-HT contains a signal peptide (SP), 22 tandem copies of a leucine-rich repeat (LRR), a transmembrane domain (TM), and a HaloTag domain. **b** Chemical structure of Alexa488-CLE9. **c** Green fluorescence of Alexa488-CLE9 detected on the outer surface of the microbeads under confocal laser scanning microscopy. Alexa488-CLE9 was added at 100 nM. Scale bar: 50 μm. **d** Loss of fluorescence by the addition of an excess (100 μM) quantity of unlabeled CLE9

To achieve high-throughput and automated chemical screening using a binding assay-based approach, we overexpressed recombinant BAM1, in which the cytoplasmic kinase domain was replaced with HaloTag in tobacco BY-2 cells (Fig. 1a). After membrane preparation and solubilization, we immobilized BAM1-HaloTag (BAM1-HT) onto HaloLink Sepharose microbeads to give BAM1-Sepharose. We also synthesized Alexa488-CLE9 by reacting Alexafluor488-NHS ester with the ε-amino group of [Lys^2^]CLE9 (Fig. 1b). The receptor-binding affinity of CLE9 has been shown to be unaffected by Leu^2^-to-Lys substitution even after modification of the ε-amino group with the functional groups^17^.

When BAM1-Sepharose microbeads were incubated with Alexa488-CLE9 at 100 nM, strong green fluorescence of Alexa488-CLE9 was detected on the outer surface of the microbeads under confocal laser scanning microscopy (Fig. 1c). This fluorescence was completely abolished by the addition of an excess quantity of unlabeled CLE9, indicating that the observed CLE9–BAM1 interaction is specific and reversible (Fig. 1d). Because this fluorescence-based assay is compatible with a 384-well microplate format, we employed this system for the large-scale chemical screening of competitive inhibitors of the CLE9–BAM1 interaction.

### Screening of specific inhibitors of the CLE9–BAM1 interaction

**Fig. 2:**
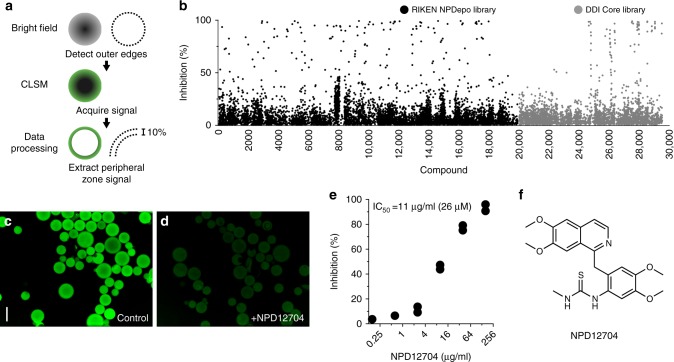
Chemical screening of specific inhibitors of the CLE9–BAM1 interaction. **a** Data acquisition algorithm used in the screening. The outer edges of the Sepharose beads were detected under a bright field. Efficiency of Alexa488-CLE9 binding to BAM1-Sepharose was determined by extracting the average signal strength per pixel in the inner peripheral zone (10% of diameter) of the beads under confocal laser scanning microscopy (CLSM). **b** Percent inhibition distribution pattern of compounds in the first round of screening. **c** Alexa488-CLE9 signals on BAM1-Sepharose observed using an Opera confocal imaging system. Alexa488-CLE9 was added at 100 nM. Scale bar: 100 μm. **d** Inhibition of Alexa488-CLE9 binding to BAM1-Sepharose by NPD12704 at 40 μg ml^−1^ (95 μM). **e** Dose–response curve of inhibition of Alexa488-CLE9 binding to BAM1 by NPD12704. **f** Structure of NPD12704

We screened 19,449 compounds from a chemical library at the RIKEN Natural Products Depository (NPDepo)^18^ and 9600 compounds from the DDI Core Library (Drug Discovery Initiative, University of Tokyo) using glass-bottom 384-well microplates, in which BAM1-Sepharose microbeads were dispensed and mixed with 100 nM Alexa488-CLE9. Fluorescent images were acquired using a PerkinElmer Opera high-throughput confocal imaging system and analyzed using a Columbus image data storage and analysis system, by which we calculated the average signal strength per pixel in the inner peripheral zone of the beads (Fig. 2a).

We searched for compounds that inhibited Alexa488-CLE9 binding to BAM1-Sepharose microbeads by more than 50% of the control at 10 μg ml^−1^ (NPDepo Library) or 20 μM (DDI Core Library). In the first round of screening, 165 compounds (0.85%) from the NPDepo Library and 81 compounds (0.85%) from the DDI Core Library passed the criteria (Fig. 2b, Supplementary Data 1). After confirming reproducibility at least two times, we obtained 15 hits (0.08%) in the NPDepo Library, but no hits in the DDI Core Library. However, 9 of the 15 hit compounds showed no clear dose-dependent inhibition, indicating that they interfere with the CLE9–BAM1 interaction in a non-competitive manner. Among the remaining six compounds, we excluded four colored compounds and one that was a frequent hit, resulting in the identification of one compound, NPD12704, as a candidate-specific inhibitor of BAM1 receptor kinase (Fig. 2c–f). NPD12704 inhibited Alexa488-CLE9 binding to BAM1-Sepharose microbeads with an IC_50_ value of 26 μM.

### NPD12704 inhibits ligand binding preferentially to BAM1

To test whether NPD12704 specifically inhibits the ligand–receptor interaction, we employed a competitive photoaffinity labeling assay using a radiolabeled photoactivatable ligand. We previously reported that [^125^I]ASA-[Ara_3_]CLV3 glycopeptide directly binds to and cross-links both CLV1 and BAM1 receptor kinases that redundantly regulate SAM stem cell populations in *Arabidopsis*^15^. We also showed that [^125^I]ASA-CLE9 binds CLV1, BAM1, and BAM2, indicating that CLE9 acts as an agonist for all CLV1, BAM1, and BAM2^17^. Their apparent binding constant (*K*_d_) was determined to be 1.0 nM for [Ara_3_]CLV3-CLV1, 1.8 nM for CLE9–BAM1, and 11.9 nM for CLE9-BAM2^14,17^. We were, therefore, interested in determining whether NPD12704 inhibits each of the specific ligand–receptor pair interactions between CLE9–BAM1, [Ara_3_]CLV3-BAM1, CLE9-CLV1, [Ara_3_]CLV3-CLV1, and CLE9-BAM2.

**Fig. 3:**
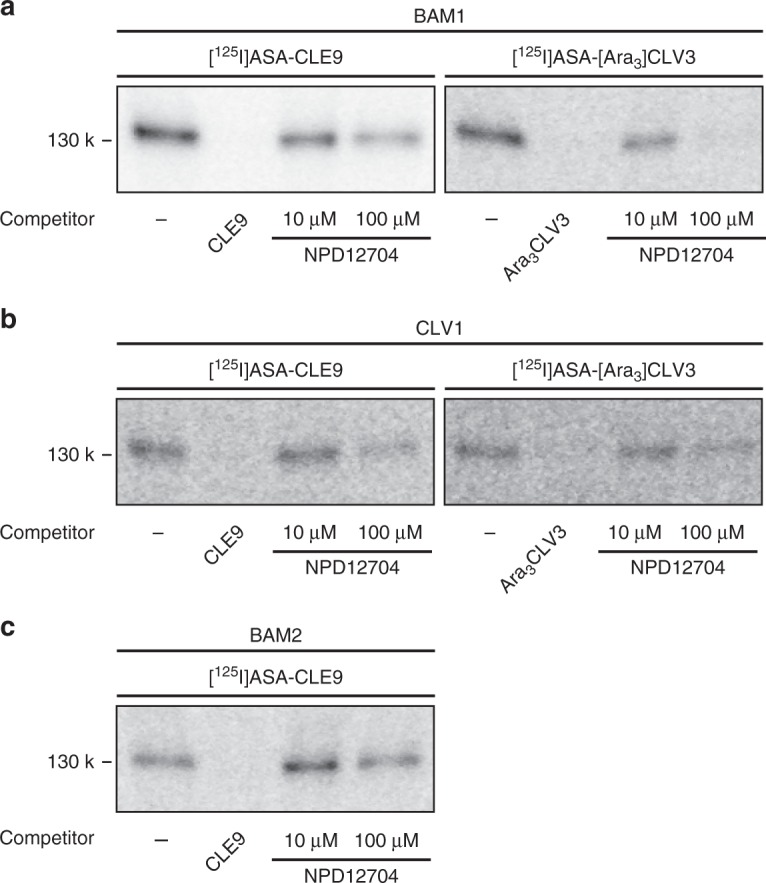
NPD12704 inhibits ligand binding preferentially to BAM1. **a** Photoaffinity cross-linking of 30 nM [^125^I]ASA-CLE9 or [^125^I]ASA-[Ara_3_]CLV3 with BAM1 in the presence of NPD12704 or various competitors. CLE9 and [Ara_3_]CLV3 were added at 10 μM. NPD12704 was added at the indicated concentrations. **b** Photoaffinity cross-linking of [^125^I]ASA-CLE9 or [^125^I]ASA-[Ara_3_]CLV3 with CLV1 in the presence of NPD12704. **c** Photoaffinity cross-linking of [^125^I]ASA-CLE9 with BAM2 in the presence of NPD12704

Photoaffinity cross-linking of [^125^I]ASA-CLE9 with BAM1 followed by autoradiography enabled us to identify the ligand–receptor complex at 130 kDa (Fig. 3a, Supplementary Fig. 1). We confirmed that this band decreased to 62% and 33% of the control by the addition of 10 μM and 100 μM NPD12704, respectively. We also observed that NPD12704 inhibited the binding of [^125^I]ASA-[Ara_3_]CLV3 to BAM1 even more strongly than it inhibited the binding of [^125^I]ASA-CLE9 to BAM1. The cross-linked band of [^125^I]ASA-[Ara_3_]CLV3 with BAM1 decreased to 46% of the control following the addition of 10 μM NPD12704 and became almost completely undetectable by addition of 100 μM NPD12704. By contrast, NPD12704 displayed no inhibition of the interaction between both the [^125^I]ASA-CLE9 and CLV1 pair and [^125^I]ASA-[Ara_3_]CLV3 and CLV1 pair at 10 μM (Fig. 3b). NPD12704 only partially prevented these interactions at 100 μM. Binding of [^125^I]ASA-CLE9 to BAM2 was not affected by NPD12704, even at 100 μM (Fig. 3c). These results indicate that NPD12704 interferes with ligand binding preferentially to BAM1.

### NPD12704 induces enlargement of the SAM through the BAM1

It has been reported that mutations in CLV1 lead to slight enlargement of the SAM in *Arabidopsis*, and this phenotype is considerably enhanced by additional loss of BAM1, indicating that CLV1 and BAM1 synergistically regulate the SAM stem cell population^15,19^. To investigate whether NPD12704 specifically affects the BAM1-mediated signaling pathway, we examined meristem size in wild-type, *clv1-101*, and *bam1-4* seedlings after treatment with NPD12704.

**Fig. 4:**
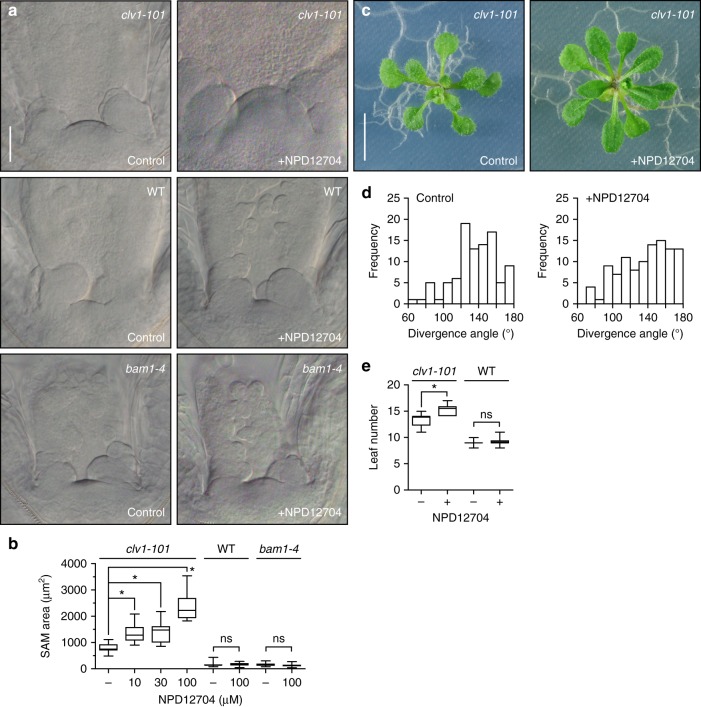
NPD12704 induces enlargement of the SAM and abnormal phyllotaxy through the BAM1 pathway. **a** Nomarski micrographs of the SAM of *clv1-101*, wild-type (WT), and *bam1-4* seedlings grown in the presence or absence of 100 μM NPD12704 for 5 d. Scale bar: 50 μm. **b** SAM size of *clv1-101*, wild-type, and *bam1-4* seedlings grown in the presence of various concentrations of NPD12704 for 5 d. Data are presented using box and whiskers plots (bottom: 25%; top: 75%; line: median; whiskers: min to max) (^*^P < 0.05, Student’s *t* test, *n* = 9–15). **c** Abnormal phyllotaxy of *clv1-101* plants cultured in the presence of 100 μM NPD12704 for 15 d. Scale bar: 5 mm. **d** Distribution of divergence angle frequencies between successive leaves in *clv1-101* cultured in the absence or presence of 100 μM NPD12704 for 15 d. **e** Rosette leaf number of *clv1-101* and wild-type plants cultured in the presence of 100 μM NPD12704 for 21 d

When *clv1-101* single-mutant seedlings were cultured in liquid medium containing 100 μM NPD12704, we observed an increase in SAM size compared with the control (Fig. 4a). NPD12704 was effective at concentrations as low as 10 μM, as judged by dose-dependence experiments (Fig. 4b). In *clv1-101* plants cultured for a longer period in the presence of NPD12704, we occasionally observed abnormal phyllotaxy accompanied by an increase in leaf number, which is often prominent in CLV-related mutants (Fig. 4c–e). By contrast, NPD12704 exhibited no visible effect on SAM size in wild-type and *bam1-4* seedlings (Fig. 4a). Leaf number was also unaffected by NPD12704 in wild-type seedlings (Fig. 4e). These results indicate that NPD12704 specifically inhibits the BAM1 pathway but only minimally inhibits the CLV1 pathway, thereby inducing SAM enlargement exclusively in the *clv1-101* mutant.

### NPD12704 suppresses CLV3-mediated root termination by blocking BAM1

In addition to its critical role in regulating SAM activity, BAM1 reportedly functions as a redundant receptor that negatively regulates cell proliferation in the root meristem through a CLE peptide-mediated pathway^20^. Loss-of-function mutations in BAM1 confer partial resistance to externally supplied non-arabinosylated CLV3 peptide in roots. Hence, we hypothesized that NPD12704 would suppress CLV3-mediated root termination by blocking the BAM1 signal transduction pathway.

**Fig. 5:**
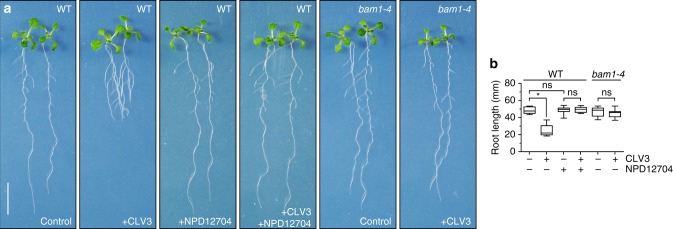
NPD12704 suppresses CLV3-mediated root termination. **a** Wild-type (WT) and *bam1-4* seedlings grown in the presence of either or both 10 nM non-arabinosylated CLV3 and 100 μM NPD12704 for 10 d. Scale bar: 10 mm. **b** Primary root length of (A) (*n* = 6–10)

When wild-type seedlings were incubated on an agar medium containing 10 nM non-arabinosylated CLV3 peptide, primary root length was reduced to 51% of that of untreated seedlings (Fig. 5a, b). At this peptide concentration, *bam1-4* roots were insensitive to CLV3 treatment, indicating that BAM1 is responsible for CLV3-mediated restriction of root growth. In contrast, when wild-type seedlings were cultured on an agar medium supplemented with 100 μM NPD12704 in addition to 10 nM CLV3, root growth was reversed to the wild-type level. NPD12704 alone did not affect root growth of wild-type plants. Based on these results, we conclude that NPD12704 functions as a specific antagonist for the receptor kinase BAM1 in planta as well as in vitro.

## Discussion

In medicinal drug development, two distinct approaches are generally employed to identify hit compounds of interest: a classical phenotype-based approach involving morphologic observations and a target-based approach that screens for direct inhibitors of critical signaling components, such as receptors or enzymes. An analysis of the origins of new medicinal drugs approved between 1999 and 2013 revealed that 70% of the novel target drugs were discovered using a target-based approach, indicating that target-based screening strategies are more prevalent than phenotype-based approaches^21^. A major advantage of the use of specific targets for screening is that the compounds can be optimized to recognize the target exclusively, thereby avoiding problematic off-target effects.

In this study, we employed a target-based receptor binding assay using a bead-immobilized receptor kinase and fluorescent-labeled peptide ligand. We screened a number of chemicals for direct interaction with the peptide hormone receptor in competition with labeled peptide ligands. This rational in vitro target-based approach returned a primary hit rate of 0.85%, with a low false-positive frequency, and eventually led to the identification of NPD12704 as a specific BAM1 antagonist. NPD12704 induces an enlarged SAM phenotype through the BAM1 pathway, without noticeable cytotoxicity. To our knowledge, this is the first identification of a functional non-peptide antagonist that binds receptor kinases competitively with the natural peptide ligands in plants.

Our solid-phase receptor-binding assay system is also readily applicable to a series of other plant receptor kinases, as their extracellular domain alone is generally sufficient for ligand binding without any co-receptors. In addition, the use of confocal laser scanning microscopy, which detects signals exclusively within the focus zone, enables the detection of fluorescent signal associated with bound ligands without the removal of unbound ligands, thereby making the system compatible with high-throughput screening using multi-well plates. Due to its simplicity and the ability to directly measure ligand–receptor interactions, this solid-phase receptor-binding assay system could also be applied, in principle, to screening for plant receptor kinase agonists.

Drug discovery generally begins with identification of small molecule hits by high-throughput screening of chemical libraries and proceeds to development of lead compounds with higher potency through the introduction of a series of structural modifications^22^. The leads can then be optimized to drug candidates by extensive examination of their efficacy and physiochemical properties. Initial screening hits, however, do not always show high-binding affinity for the targets, as exemplified by the research history of non-peptide antagonists that block receptors for gonadotropin-releasing hormone^23^. Despite low-micromolar IC_50_ values of the initial hits, optimization by medicinal chemistry efforts ultimately led to development of potent candidates exhibiting excellent nanomolar affinities. In this context, identification of NPD12704 provides a good starting point from which additional stages in the agrochemical engineering process, such as optimization of binding affinity and target specificity, can follow.

The extracellular domain of BAM1 is comprised of 22 consecutive leucine-rich repeats (LRRs). Using photoaffinity labeling followed by chemical and enzymatic digestion, we previously showed that BAM1 interacts with [^125^I]ASA-CLE9 within the Ile^206^–Lys^261^ region, which corresponds to the LRR6 to LRR8 region relatively distal to the transmembrane domain^17^. Multiple sequence alignment and homology modeling indicated that the inner concave side of LRR6-8 of CLV1/BAM family LRR-RKs deviates slightly from the common LRR consensus and thus likely acts as a specific ligand-binding domain. Supporting this hypothesis, the *clv1-4* mutant carries a missense mutation on the inner concave side of LRR6, and introduction of the corresponding mutation in BAM1 resulted in complete loss of ligand-binding activity^17^. Recent crystallographic studies of TDR, a receptor for the CLE family peptide TDIF, further corroborate this ligand recognition model of BAM1^24,25^. In our study, NPD12704 interfered with ligand binding to BAM1 in an apparently competitive manner with the ligands. Although further studies such as crystallographic analyses are needed, it is tempting to speculate that the NPD12704 target site resides within, or close to, the LRR6-8 region of BAM1.

Over the past 20 years, more than a dozen secreted peptide hormones and their receptors have been identified in plants^1–4^. Peptide hormones directly bind to specific receptors on the cell surface, where these physicochemical interactions are converted into physiologic responses that activate downstream signaling cascades to modulate cellular processes. As the number of peptide hormones now exceeds the number of classical plant hormones, the next stage will be to explore novel and unique applications of peptide signaling systems in agriculture and food production. It has been reported, for example, that nematode-secreted peptides function as molecular mimics of endogenous plant peptides to promote parasitism^26,27^. Chemical interference of peptide hormone receptors targeted by such plant-parasitic nematodes may offer an alternative approach to control nematode infection. Our solid-phase screening system that enables high-throughput identification of peptide hormone receptor antagonists or agonists, preferably followed by structural fine-tuning, holds enormous potential for use in both practical applications and fundamental studies of plant growth and development.

## Methods

### Expression of BAM-HT and CLV1-HT in tobacco BY-2 cells

To overexpress BAM1-HT in tobacco BY-2 cells, we amplified the genomic fragment of *BAM1* corresponding to the Met^1^ to Ile^700^ region and the cDNA fragment of HaloTag (Promega), which contains the entire open-reading frame of HaloTag, by PCR. These two fragments were cloned in translational fusion using three-component ligation into the *Bam*HI/*Sac*I-digested binary vector pBI121 using an In-Fusion HD Cloning kit (Clontech). CLV1-HT and BAM2-HT were prepared as previously described ^13,17^. Transformation of tobacco BY-2 cells and preparation of microsomal fractions were described previously^13^.

### Preparation of Alexa488-CLE9 and photoactivatable [^125^I]ASA-CLE9

The Fmoc-protected CLE9 analog Fmoc-[Lys^2^]CLE9 was synthesized by Fmoc chemistry using a peptide synthesizer (Biotage Initiator + Alstra). Alexa Fluor 488 succinimidyl ester (0.3 mg, Thermo Fisher), Fmoc-[Lys^2^]CLE9 (3.1 mg), and NaHCO_3_ (10.0 mg) were dissolved in 250 μl of 50% acetonitrile and stirred for 1 h in the dark at room temperature. Crude peptide was purified by reverse-phase HPLC and lyophilized to yield analytically pure Fmoc-[(Alexa488)Lys^2^]CLE9. To this purified peptide, 200 μl of 20% piperidine was added in acetonitrile, followed by incubation for 1 h in the dark at room temperature. This deprotected peptide was further purified by reverse-phase HPLC and lyophilized to obtain analytically pure [(Alexa488)Lys^2^]CLE9 (Alexa488-CLE9). [^125^I]ASA-CLE9 and [^125^I]ASA-[Ara_3_]CLV3 were prepared as previously described^15,17,28^.

### Covalent immobilization of BAM1-HT onto microbeads

Aliquots (350 mg proteins) of microsomal fractions of BY-2 cells overexpressing BAM1-HT were solubilized in 35 ml of solubilization buffer (20 mM HEPES–KOH [pH 7.5], 150 mM KCl, and 1% Triton X-100). Solubilized samples were centrifuged at 100,000 × *g* for 30 min at 4 °C. HaloLink resin (1.5 ml, Promega) was added to the resultant supernatant and rotated gently for 16 h at 4 °C, followed by washing with 35 ml of solubilization buffer. BAM1-HT–immobilized beads were re-suspended in 1.5 ml of binding buffer (50 mM MES–KOH [pH 5.5] and 0.1% Triton X-100). For visualization of on-bead ligand–receptor interaction by confocal microscopy, aliquots (5 µl) of BAM1-Sepharose microbeads were suspended in 20 µl of binding buffer containing 100 nM Alexa488-CLE9, followed by incubation for 30 min on ice. Bound Alexa488-CLE9 was directly visualized using a confocal laser scanning microscope (Olympus FV-300) with excitation at 488 nm and emission at 505–525 nm. Non-specific binding was assayed by adding a 100-fold excess of unlabeled CLE9 as a competitor.

### High-throughput screening

Compound libraries were provided by the Drug Discovery Initiative (DDI), University of Tokyo (9,600 compounds), and RIKEN NPDepo (19,449 compounds). BAM1-Sepharose microbeads were suspended in binding buffer at a density of 15 μl packed volume per ml and dispensed into wells of a 384-well glass-bottom plate (PerkinElmer ViewPlate-384) at 10 μl per well using a single-line dispenser (Biotec mini-Gene LD-01). Alexa488-CLE9 and chemical compounds were then added to the plates using an automatic pipette multi-dispenser (Biotec EDR-384). Final concentrations of Alexa488-CLE9, NPDepo Library, and DDI Core Library compounds were 100 nM, 10 μg ml^−1^, and 20 μM, respectively. The plates were incubated for 5 min at room temperature. In order to capture Alexa488-CLE9 fluorescence signals on BAM1-Sepharose, we used Opera (PerkinElmer OperaQEHS) and the Opera-TwisterII-iLinkPro system (PerkinElmer TwisterII, iLinkPro Ver.1.03). Images were acquired with Opera using a 10 × objective and the following channels: Ex1Cam1 using 488-nm laser excitation and 450/50-nm emission filters for bright fields and Ex1Cam2 using 540/75-nm emission filters for the Alexa488-CLE9 fluorescence signal. The images were analyzed using Columbus software (PerkinElmer). The outer edges of the Sepharose beads were detected using a bright-field image. The efficiency of Alexa488-CLE9 binding to BAM1-Sepharose was determined by extracting the average signal strength per pixel in the inner peripheral zone (10% of diameter) of the beads.

### Photoaffinity labeling

Aliquots of microsomal fractions (600 μg proteins) expressing BAM1-HT, CLV1-HT, or BAM2-HT were suspended in 250 μl of binding buffer (50 mM MES–KOH [pH 5.5] with 100 mM sucrose) containing 30 nM [^125^I]ASA-CLE9 or [^125^I]ASA-[Ara_3_]CLV3 in the presence or absence of various concentrations of NPD12704 or competitor peptides and then incubated for 20 min on ice. Bound and free ligands were separated by layering the reaction mixture onto 900 μl of wash buffer (50 mM MES–KOH [pH 5.5] with 500 mM sucrose) and centrifuging for 5 min at 100,000 × *g* at 4 °C. After discarding the supernatant, the pellet was irradiated on ice for 20 min with a UV lamp (model ENF-260C/J [365 nm]; Spectronics) at a distance of 1 cm. Cross-linked membrane proteins (600 μg) were solubilized with 400 μl of solubilization buffer (20 mM HEPES–KOH [pH 7.5], 150 mM KCl, and 1.0% Triton X-100). The lysate was kept on ice for 30 min and then centrifuged for 30 min at 100,000 × *g* at 4 °C to remove insoluble material. The supernatant was then incubated for 1 h with 2 μg aliquots of anti-HaloTag antibody (Promega) at 4 °C. The resulting immune complexes were immunoprecipitated with 50 μl aliquots of Protein A-Sepharose (GE Healthcare) for 1 h at 4 °C. Immunoprecipitated proteins were separated by SDS-PAGE on a 7.5% acrylamide gel. Gels were dried, exposed to a bioimaging plate (MS 2025; Fujifilm) for 3 days at room temperature, then analyzed using an imaging plate reader and bioimaging analyzer (FLA-9000; Fujifilm).

### Phenotypic assay

For SAM observation, surface-sterilized *Arabidopsis clv1-101* and *bam1-4* mutant seeds or wild-type seeds (≈20 seeds) were directly sown into 1.0 ml of B5 medium containing 1.0% sucrose in the presence of various concentrations of NPD12704 in 24-well microplates, then incubated without shaking under continuous light at 22 °C. After 5 days, seedlings were fixed with acetic acid–ethanol (1:9), cleared in a mixture of chloral hydrate, glycerol, and water (8:1:2, w/v/v), and then observed under a microscope equipped with Nomarski optics (BX-50; Olympus). All images were taken at the focal plane corresponding to the median optical section of the SAM. SAM area was measured on a median plane by calculating the area above the straight line between the basal edges of two opposite leaf primordia. All areas were measured using ImageJ software. For observation of leaf phyllotaxy patterns, plants were grown on medium solidified with 0.7% agar in the absence or presence of 100 µM NPD12704. All plants were grown under continuous light at 22 °C. Photographs of plants were taken at 15 days after germination (DAG), and divergence angles were measured using ImageJ software. For observation of root growth, plants were grown vertically on medium solidified with 1.5% agar in the absence or presence of 10 nM non-arabinosylated CLV3 and/or 100 μM NPD12704 under continuous light at 22 °C. Photographs of plants were taken at 10 DAG, and the length of primary roots was determined.

### Reporting summary

Further information on experimental design is available in the Nature Research Reporting Summary linked to this article.

## Supplementary information

Supplementary Information

Reporting Summary

Supplementary Data 1

Descriptions of Additional Supplementary Files

## Data Availability

The datasets generated during and/or analyzed during the current study are available from the corresponding author on reasonable request.
