# Supplementary figures and images for: Screening and identification of a non-peptide antagonist for the peptide hormone receptor in Arabidopsis
Source: Commun Biol. 2019 Feb 15;2:61. doi: 10.1038/s42003-019-0307-8 (PMC6377654; doi:10.1038/s42003-019-0307-8)

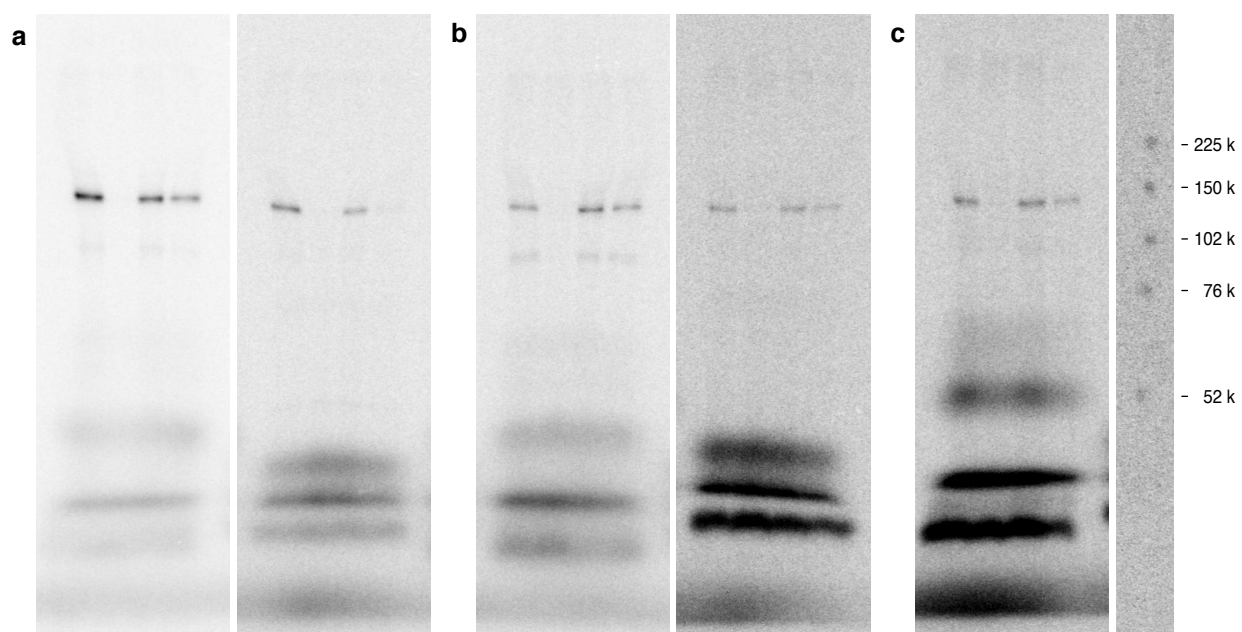

**Supplementary Fig. 1.** The full-length unedited blot images for Fig. 3a–c.

Supplement: Supplementary file 1 — Supplementary Information [file 42003_2019_307_MOESM1_ESM.pdf]
