# Supplementary material for: Screening and identification of a non-peptide antagonist for the peptide hormone receptor in Arabidopsis
Source: Commun Biol. 2019 Feb 15;2:61. doi: 10.1038/s42003-019-0307-8 (PMC6377654; doi:10.1038/s42003-019-0307-8)
Supplement: Supplementary file 4 — Descriptions of Additional Supplementary Files [file 42003_2019_307_MOESM4_ESM.doc]

Descriptions of Additional Supplementary Files

**File Name:** Supplementary Data 1

**Description:** The source data underlying the graphs presented in the main figures. The source data for Fig. 2b, 2e, 4b, 4d, 4e and 5b are shown.
